# My Diabetes & Me: study protocol for a randomised controlled trial to test the clinical and cost-effectiveness of a diabetes self-management education programme for adults with intellectual disabilities.

**DOI:** 10.3310/nihropenres.13964.1

**Published:** 2025-10-06

**Authors:** Laurence Taggart, Gary McDermott, Alison Dunkley, Maria Truesdale, Vivien Coates, Kamlesh Khunti, Mike Clarke, Dan Pollard, Alan Brennan, Michelle Hadjiconstantinou, Molly Caba, Colette Jackson, Aimee Anderson Smyth, Cliona McDowell, Ashley Agus, Sorcha Toase, Janet Schofield, Rosie Kelly

**Affiliations:** 1 Queen's University Belfast, Belfast, Northern Ireland, UK; 2 University of Leicester, Leicester, England, UK; 3 University of Glasgow College of Medical Veterinary and Life Sciences, Glasgow, Scotland, UK; 4 Ulster University - Magee Campus, Londonderry, Northern Ireland, UK; 5 Northern Ireland Clinical Trials Unit, Belfast, UK; 6 University of Sheffield, Sheffield, UK; 7 Compass Advocacy Network Limited, Ballymoney, Northern Ireland, UK

**Keywords:** Keywords: intellectual disability, type 2 diabetes, self-management, education programme, HbA1c, randomised controlled trial.

## Abstract

**Background:**

International guidelines recommend structured diabetes education to empower individuals with type 2 diabetes (T2D). While DESMOND is an effective programme for T2D management, it is often inaccessible to people with intellectual disabilities (ID) due to their unique needs. There is limited evidence on the effectiveness of adapted T2D education for this group, despite the importance of tailored support in preventing complications and early mortality.

We previously adapted the DESMOND programme for adults with ID, creating DESMOND-ID. A feasibility study showed it is possible to recruit and deliver the programme to adults with ID and their carers, who found it valuable. Initial findings suggest DESMOND-ID may improve blood glucose control, warranting further investigation through a large-scale randomised controlled trial (RCT).

**Methods:**

The "My Diabetes & Me" study will be conducted in two stages: an internal pilot and a main RCT. The pilot will recruit 108 participants over 10 months to assess recruitment and retention, using glycated haemoglobin (HbA1c, mmol/mol) at six months as the primary outcome. This will inform the design of the main study.

Across both stages, 450 participants will be randomly assigned to receive either the DESMOND-ID intervention or treatment as usual (TAU). The intervention group, with their carers, will attend weekly sessions for seven weeks, plus two booster sessions at one and three months post-programme.

Primary outcome is HbA1c at six months. Secondary outcomes include HbA1c at 12 and 18 months (pilot only), anthropometric data, self-reported outcomes, and other risk factors. A process evaluation will explore barriers and facilitators to implementation using qualitative and quantitative methods.

**Conclusion:**

DESMOND-ID is the first structured T2D education programme tailored for adults with ID, and this RCT is the first to evaluate its clinical and cost-effectiveness.

**Trial Registration:**

09/11/2022

ISRCTN83150600 ( https://doi.org/10.1186/ISRCTN83150600)

## Plain English Summary

People with type 2 diabetes (T2D) are encouraged to take part in education programmes to help them understand their condition and manage it well. One such programme, called DESMOND, has been shown to work.

However, many people with intellectual disabilities (ID) cannot take part because these programmes are not designed for their needs. This matters because without the right support, people with ID are at higher risk of serious health problems.

To address this, we adapted DESMOND to create a version specially designed for adults with ID and their carers, called DESMOND-ID. In an earlier, smaller study, we found that DESMOND-ID could be delivered successfully and was well received. People told us it was useful, and there were early signs that it may help improve blood sugar levels.

We are now planning a large UK-wide study, called My Diabetes & Me, to properly test whether DESMOND-ID works and whether it provides good value for money. This study has two parts:

•   A pilot involving 108 people, to check if the study can run smoothly and to track their blood sugar levels over six months.

•   A larger main study including 450 people in total (counting those in the pilot). Half of the participants will take part in DESMOND-ID, while the other half will receive their usual diabetes care.

The DESMOND-ID programme includes seven weekly sessions followed by two review sessions, delivered to people with ID and their carers. We will measure blood sugar levels and other health indicators to see if the programme improves health. We will also study how it is delivered in different settings, and what helps or makes it harder to run.

This will be the first large study of a diabetes self management education programme designed specifically for people with intellectual disabilities.

## Background

Worldwide the International Diabetes Federation (IDF) estimates that 589 million people have diabetes and more than 90% of these people have type 2 diabetes (T2D) ^1^. According to the World Health Organisation (WHO), T2D is increasing in the general population, bringing significant health risks, and rising costs for healthcare providers globally ^2^. According to the National Institute for Health and Care Excellence [NICE], management of T2D aims to optimise blood glucose levels (HbA1c) to avoid long-term complications (e.g. retinopathy, foot problems, kidney disease, peripheral and autonomic neuropathy, and cardiovascular disease) and earlier and avoidable death ^3^. Individuals living with T2D require considerable self-care skills and practices to self-manage their condition, and avoid negative health behaviours in relation to diet, physical activity, BMI, blood pressure, smoking, alcohol consumption, and medication adherence ^3^. However, some find this difficult and diabetes self-management education (DSME) programme ^4^.

DSME programmes can help improve self-care behaviours, quality of life, lower HbA1c levels and impact disease progression ^5^. Diabetes education has been shown to be cost-effective by reducing hospital admissions and lowering lifetime healthcare costs through a reduced risk of complications ^6, 7^. Within the UK, the National Service Framework for Diabetes: Standards ^8^, the NICE guidelines on T2D ^3^ and the UK’s Department of Health guidance ^9^, all recommended that everyone with T2D should be offered the opportunity to attend a DSME programme. The most established and robustly evaluated, DSME programme implemented across the UK is DESMOND ^10^.

DESMOND (Diabetes Education and Self-Management for Ongoing and Newly Diagnosed) is a 6-hour group education programme, delivered by health professionals and lay educators over two half days or one full day. DESMOND (and similar DSME programmes) have been found to improve individuals’ understanding of T2D, improve diet, increase physical activity, reduce weight, help effective smoking cessation; reduce depression, and promote positive behaviour change, thereby improving cardiovascular disease risk factors (blood pressure, blood lipids, cholesterol) and decreasing glycated haemoglobin (HbA1c) levels, with sustained improvements maintained at 12-months and three years ^10– 13^. DESMOND is a is a cost-effective educational intervention in the management of T2D ^6, 14^.

Since the original DESMOND initiative for people diagnosed with T2D, it has been successfully adapted to meet the needs of underserved populations and those who are at increased risk of T2D, including those from ethnic minority communities ^15, 16^, people with T2D and co-morbidities ^17^, those at risk of diabetes ^18^, and women with gestational diabetes ^19^. A fundamental goal of DESMOND is a more engaged and empowered individual, whereby the person living with diabetes becomes the expert. The robust evidence of the clinical and cost-effectiveness of the DESMOND education programmes have strongly highlighted the importance of structured group education to support T2D self-management systematically. The roll-out of DESMOND across the UK has enabled healthcare providers to meet many of the demands of people with T2D, but it had not been offered to, or adapted for, people with ID ^20– 22^ before our earlier work ^23^.

The American Association for Intellectual and Developmental Disabilities define an ID as:

*‘A disability characterised by significant limitations in both intellectual functioning and adaptive behaviour as expressed in conceptual, social, and practical skills. This disability originates during the developmental period, which is defined operationally as before the individual attains age 22.’* ^24^

Intellectual functioning relates to intelligence, alongside the belief that it is influenced by human functioning dimensions and systems of support ^24^. Whilst adaptive behaviour includes conceptual skills (e.g., language and literacy, self-direction), social skills (e.g., interpersonal skills, ability to follow rules) and practical skills (e.g., personal care, use of money/phone) that have been learned and are performed by individuals in their daily lives ^25^. To explain the wide range of different abilities the idea of a continuum of disability has been used from mild, moderate, severe and profound ID.

Within the UK approximately 1.3 million adults (18+ years) are said to have an ID ^26^. People with ID are now living longer and developing more chronic physical health conditions such as diabetes ^27^. T2D is 2–3 times more common in people with ID than the general population ^20, 21^, and this population have poorer outcomes including delayed diagnosis, poor management of symptoms, more severe complications, unnecessary hospitalisations, and premature mortality. Furthermore, they are not offered the DSME programmes recommended for, and routinely available to the general adult population ^20– 22^.

The Equality Act 2010 ^28^ in the UK sets out the legal requirement for public health services to provide reasonable adjustments for people with a disability, which should include provision of accessible therapeutic and educational support. Addressing this gap is a UK NHS priority to avoid premature deaths and serious diabetic complications, to improve healthcare among people with ID and to reduce costs for healthcare providers. Yet if people with ID and T2D are not offered diabetes education programmes, they cannot benefit from the potential health improvements that have been found for other populations.

Current national UK diabetes education programmes, and other structured diabetes education programmes internationally, have not been tailored to address the needs of people with ID, which include their cognitive and communication needs, low literacy skills and learning styles ^20– 22^. This is despite the UK’s NICE guidelines for T2D management ^3^ recommending that diabetes education should meet the specific needs of different populations. Evidence of the impact of structured diabetes education programmes targeting HbA1c for adults with ID is sparse. We aim to fill this gap by evaluating a structured T2D education programme for adults with ID and to determine if it is clinically and cost-effective compared to TAU.

During our recent Diabetes UK feasibility study ^23^, we successfully adapted and tested DESMOND for use with adults with ID (creating the DESMOND-ID programme). DESMOND-ID is delivered over 7 weeks (2 hours per week), where the adult with ID is accompanied by a family/paid carer or spouse/partner or supporter, where available, followed by two booster sessions at one and three months later. Our feasibility study has also shown that DESMOND-ID can be delivered to adults with ID and T2D in the context of a randomised trial ^23^. In addition, it is possible to identify and recruit adults with ID and T2D into such a study, obtain informed consent, obtain blood samples, administer questionnaires, and randomise the adults to either the intervention or TAU. The feasibility study suggests that DESMOND-ID may be able to decrease HbA1c levels. In addition, DESMOND-ID was valued and accepted by adults with ID, carers and educators. Results were promising but need confirming in a definitive randomised trial.

Therefore, the aim of this prospective study is to determine whether DESMOND-ID is effective towards management of HbA1c and cost-effective compared to TAU in adults with ID and T2D. We will test if DESMOND-ID can bring about the same benefits for adults with ID as the DESMOND studies have shown for adults in the general population, improving the health of people living with T2D and ID, and reducing inequalities in health.

## Objectives

1. To conduct a UK-wide, multicentre randomised trial to determine the effectiveness of DESMOND-ID on improving HbA1c levels (primary outcome) and a range of secondary outcomes compared to TAU.
2. To determine the cost-effectiveness of DESMOND-ID compared to TAU via a within-trial economic evaluation and a long-term model.
3. To determine the facilitators, barriers and mechanisms of actions involved in the DESMOND-ID process via a process evaluation.

## Methods and design

### Patient and Public Involvement (PPI)

Our PPI partners for this study are Compass Advocacy Network (CAN) a non-profit organisation in Northern Ireland who work with work with children and adults with learning disabilities, autism and mental health issues. Staff and service users at CAN have been involved with the DESMOND-ID programme since our original feasibility testing phase in 2018 ^23^. Feedback obtained from our PPI partners during this phase enabled us to refine the DESMOND-ID content and delivery based upon the unique healthcare challenges experienced by adults with learning disabilities, prior to the commencement of our RCT.

In preparation for this RCT our PPI partners at CAN helped us to develop and test user-friendly participant information sheets adapted to meet the comprehension and communication needs of adults with learning disabilities. In addition, they led on the development of a suite of promotional materials, such as videos and posters, to help support participant recruitment.

We will work with our PPI partners throughout the study to develop a suitable dissemination plan, ensuring findings from our study reach a wide ranging audience. We will work with our PPI partners to refine the data to ensure that the results are formatted and presented in a manner that is suitable for adults with learning disabilities and their advocates.

### Study design

The My Diabetes and Me study is a 2-stage parallel group randomised trial with an internal pilot, economic evaluation and process evaluation. The internal pilot (Stage 1) will run for 10 months to assess recruitment rates and retention for the primary outcome at 6 months. The internal pilot will also allow us to identify any key difficulties and address them in preparation for the main study. The first three clinical sites will recruit a total of 108 adults with ID and T2D during this period. Participants enrolled in the pilot will be included in the analysis of the main study. We will use Avery *et al.,* ^29^ traffic light system to guide our progression criteria as recommended in recent best practice. This protocol is reported in accordance with the Standard Protocol Items: Recommendations for Interventional Trials (SPIRIT) guidelines ^30^.

### Study sample

In total, 191 participants in each randomised group will have 90% power to detect a difference in means of 0.5% in HbA1c (mmol/mol) at 6 months. This 0.5% cut-off point is accepted in the diabetes community as being a minimal clinically important difference, ensuring that an effect of this size or greater will be seen as a meaningful indication of the effectiveness of the intervention ^5^. We assumed a standard deviation of 1.5% ^31^ and used a two-group t-test with a 0.05 two-sided significance level. Assuming a dropout rate of 15%, the sample size required is 450 participants (225 per group). This dropout rate is likely to be an overestimate because our feasibility study showed that <10% of recruited adults with ID dropped out ^23^ and other National Institute for Health and Care Research (NIHR) ID intervention studies have reported similar results ^32, 33^.

The current intention is to seek an excess of 30%–40% individuals within the ID and T2D population over this sample size who might be willing to take part in the study. Therefore, 585–630 adults with ID and T2D will be identified to provide some flexibility for those not meeting the criterion, refusals and dropouts. This 30%–40% is a generous estimate and is based on our feasibility study’s recruitment rates ^23^ and other diabetes and ID studies ^34^.

### Ethical and research governance approval

Ethical approval was received by the Office for Research Ethics Northern Ireland (ORECNI: 22/NI/0156), and research governance was obtained from all participating health boards in Northern Ireland, Scotland and England.

### Status and timeline of the study

The total duration of the study will be 45 months, including follow up at 6 and 12 months after randomisation for all participants, and at 18 months after randomisation for those participants recruited to the internal pilot. We will open the first site within five months and aim to have eight sites open within 22 months. The internal pilot will run during months 5–14. Following successful confirmation of recruitment rates, the internal pilot will move into the main study. The total recruitment period will last until month 25. There will be 3 months at the end for final data analysis, reporting and close-down.

We have chosen eight clinical sites across three UK countries (Northern Ireland, Scotland, and England) to maximise recruitment across a large population and improve the applicability of our results. The clinical sites cover a range of urban and semi-rural areas, and include areas with high levels of deprivation, a wide range of ethnicities and are culturally diverse. We will use a range of community settings to deliver the DESMOND-ID education programme. To be involved in the study, community ID or other appropriate healthcare professionals at the clinical sites must be prepared to participate in the DESMOND-ID training package and be prepared to deliver the intervention.

### Recruitment process

Recruitment between January 2023 and December 2024 will take a multi-pronged approach. Potential participants with ID and T2D will be identified via their community ID teams and associated services (including ID residential homes and ID-day centres/opportunities). While additional potential participants with a mild/moderate ID and T2D who might not be known to statutory ID services will be identified by primary care/general practices in each site using the Read Code system. Each ID clinical team will work with us to compile a list of those who have already been contacted to avoid duplication. Each community ID team and GP practice will send out an invitation letter, participant information sheet (PIS) and reply slip. This will be followed by a telephone call and those who agree will be referred to the study by the clinicians in the participating clinical sites who will undertake the initial screenings.

We will also run a social media campaign to advertise the study and help with further identification of potentially eligible participants. The study will also be advertised in settings where adults with ID attend (e.g., day centres and care providers). If a potential participant indicates their willingness to join the study, their contact details will be forwarded to the team. 

The community ID team/GP Practice/housing provider/other ID service provider will send the potential participant an initial invitation letter with a reply slip. The community ID nurses at each clinical site will telephone those who have not returned their reply slip to remind them and support them to make an informed decision about participating in the study. When reply slips are returned to the research team, a clinical research nurse will telephone the participant to arrange to visit and discuss the study with interested participants in the presence of a family or paid carer or spouse/partner/advocate as appropriate.

All potential participants will be screened against the inclusion and exclusion criteria. All screening data will be recorded onto the screening log. The outcome of the screening process and reasons for the non-recruitment of potentially eligible participants will be recorded on the My Diabetes and Me screening log.

We know that some adults with an ID and T2D will be living alone in their own home and may not have a carer to accompany them during the education programme, and we will not exclude those individuals from taking part in the study.

### Inclusion criteria

Participants will be eligible to participate in the study in accordance with the following criteria:

- Mild/moderate ID as confirmed by health professional/medical records
- Diagnosed with T2D
- Aged ≥18years
- Have sufficient English language skills to fully consider/give consent, and be able to speak and understand English to undertake the programme if allocated to the intervention.
- Living in the community.

### Exclusion criteria

- Type 1 Diabetes
- Severe/profound ID
- Displaying severe challenging behaviour
- Acute psychotic illness
- Lack mental capacity to give consent.

### Consent

Written informed consent from each participant with ID and T2D will be obtained by clinical research nurses and/or research associates before entry into the study. According to the UK Mental Capacity Act 2005 ^35^ it is often wrongly assumed that all people with ID do not have the mental capacity to make decisions of their own. It must be assumed that an adult with an ID has the capacity until proven otherwise. Therefore, it is important that the person is given the information required in a user-friendly format to make an informed decision using reasonable adjustments. We plan to manage on-going consent including assessment of capacity to consent using the following steps.

The research team and clinical research nurses will receive study specific training at each site in how to assess capacity to consent and ensure informed consent is maintained throughout the project on a case-by-case basis. All participants will receive an easy read PIS and consent form with pictures and symbols to explain the purpose of the study and what is involved. The PIS and consent form will be prepared in collaboration with our PPI representatives. The research team and clinical research nurses will clearly explain the decisions to be made about joining the study, having bloods and anthropometric measurements taken, and completing questionnaires at several time points, and being randomised to either the intervention or TAU group. The research team and clinical research nurses will explain what is involved in participating in the DESMOND-ID diabetes education programme (time commitments) if randomised to that group or what will happen if randomised to the control group.

The study team will assess if the person can retain the information and weigh up the pros and cons of making the decision to participate in study, allowing for plenty of time to communicate with the person with ID/T2D and to check they can retain this information. Where needed, the research team and clinical research nurses will ask for a family member, carer, partner or advocate who is familiar with the communication needs of the person with ID/T2D to be present during the interview.

### Randomisation and treatment allocation

When informed consent has been obtained participants will be randomised by the Northern Ireland Clinical Trials Unit (NICTU) using a centralised randomisation system in a 1:1 ratio, with allocation concealed until the person joins the study. Randomisation will be completed by an appropriately trained and delegated member of the NICTU team. Participants will be allocated to the intervention or TAU group. Randomisation will be stratified by site. At the time of randomisation, each participant will be allocated a unique alphanumeric code which will be used throughout the study for participant identification. The research team will then ensure that the participant’s GP is informed of their participation in the study. Due to the nature of the intervention, it is not possible to blind participants to their allocated treatment.

### Study intervention

DESMOND/DESMOND-ID is based on a series of psychological theories of learning and education: Leventhal’s Common-Sense Theory (i.e. illness representation, illness beliefs ^36^), Dual Process Theory (process of learning ^37^), and Social Learning Theory (i.e. self-efficacy ^38^). The philosophy of the programme was founded on the empowerment of individuals living with diabetes, as evidenced in published work ^39^ and its development followed a systematic approach, guided by the MRC framework for developing and evaluating complex interventions ^40, 41^.

In My Diabetes and Me, the DESMOND-ID programme will be delivered face-to-face in a range of community settings over a period of 7 weeks to 6–8 adults with ID and T2D and their carer/partner/advocate in a group setting, with two booster sessions in subsequent months (1 and 3 months after the end of the weekly sessions). The education sessions will be delivered by two healthcare professionals in each site, who will receive 3 days of training in DESMOND-ID education programme, plus an additional day of training before delivery of the booster sessions. The healthcare professionals will also be allocated 3 days for preparation and supervision of the delivery of the intervention. Staff from the DESMOND-ID team will conduct several quality assurance checks at each site throughout the duration of delivery.

Week 1 of the programme focuses on carers/partners/advocates only with the aim of improving their understanding of T2D and how DESMOND-ID works along with their supporting role. In weeks 2–7, we encourage the adult with ID/T2D and their carer/partner/advocate to attend together if possible. These weeks focus on: introductions to ‘My story with T2D, My body and T2D; What is T2D; What does T2D do to your body; Food and blood sugar, Knowing what your blood sugar levels mean; Being active; Heart and circulation problems; Other T2D health problems; What can I do to keep healthy; Food and fats; Making healthier food choices; and Developing a diabetes health action plan’ (see Table 1).

**Table 1.** DESMOND-ID structured education programme session plan.

Week 1	Week 2	Week 3	Week 4	Week 5	Week 6	Week 7
Carers and advocates only session.	Welcome and introduction (25 mins)	Welcome back (20 mins)	Welcome back (20 mins)	Welcome back (20 mins)	Welcome back (20 mins)	Welcome back (20 mins)
	My story with diabetes (part 1) (15 mins)	My story with diabetes (part 2) (15 mins)	Knowing what your blood sugar levels mean (35 mins)	Heart and circulation problems: what can I do to keep healthy? (part 1) (40 mins)	Food and fats (35 mins)	Diabetes health action plan: what will I work on? (35 mins)
	My body and diabetes (20 mins)	What diabetes does to your body? (25 mins)	Break (15 mins)	Break (15 mins)	Break (15 mins)	Break (15 mins)
	Break (15 mins)	Break (15 mins)	Being active (40 mins)	Other diabetes health problems: what can I do to keep healthy? (part 2) (35 mins)	Making healthier food choices (40 mins)	Keeping my plan going (35 mins)
	What is diabetes? (35mins)	Food and blood sugar (35 mins)	What did I learn today? (10 mins)	What did I learn today? (10 mins)	What did I learn today? (10 mins)	Important questions and celebration of achievement (15 mins)
	What did I learn today and preparing for next week? (10 mins)	What did I learn today and preparing for next week? (10 mins)	What did I learn today and preparing for next week? (10 mins)	What did I learn today and preparing for next week? (10 mins)	What did I learn today and preparing for next week? (10 mins)	
	**Total duration:** 2 hours	**Total duration:** 2 hours	**Total duration:** 2 hours	**Total duration:** 2 hours	**Total duration:** 2 hours	**Total duration:** 2 hours

The DESMOND-ID programme has been adapted by lengthening the timings of the structured programme, simplifying the core concepts, and making greater use of pictorial representations (photos, pictures, symbols) to meet the learning needs of adults with ID. It also makes use of repetitious learning/interactive sessions, there is a strong focus on developing skills and promoting “self-efficacy” in food choices and increasing physical activity, with the involvement of carers to support the person with ID/T2D, using health action plans and goal setting which are reviewed each week, and emphasising celebration and fun.

The booster sessions will be delivered 1 and 3 months after the completion of the DESMOND-ID programme. Each booster session will also last 2 hours and will be delivered by the same trained educators. These sessions will explore how each adult with ID and T2D, and their carer/partner/advocate are implementing their health action plan and any potential barriers. Those in the DESMOND-ID group will also receive TAU, ensuring that they do not lose any treatments or care that are standard.

### Treatment as usual group

Adults with ID/T2D who are randomly allocated to the control group will receive TAU and a user-friendly book previously developed to support adults with ID to manage their T2D. TAU will be established at the start of the study and again at the end of the study. Adults in the control group will not be offered any form of diabetes group education. TAU for this population normally includes health centre visits every 3–6 months in which the person meets with their primary healthcare team. We will monitor TAU for this population across the different countries.

### Outcome measures

In both randomised groups, participants with ID and T2D will complete all questionnaires and study outcome measures at baseline, 6 and 12 months follow-up, and those in the internal pilot study will also be followed-up at 18 months.

### Lifestyle and demographic information

1. Living status - participants will be asked questions to determine their living status (where and who they live with, and any carer support they receive).
2. Medical history - a health history questionnaire will be administered to determine any pre-existing medical conditions. In addition, current medication will be recorded including dosage and frequency.

### Primary outcome measure

The primary outcome is change in HbA1c level (mmol/mol), from baseline to 6 months post-randomisation. The HbA1c blood test reflects average blood glucose levels over the last 2 to 3 months. HbA1c (mmol/mol) will be assessed by taking a venous blood sample from participants.

### Secondary outcome measures

**Figure 1.**
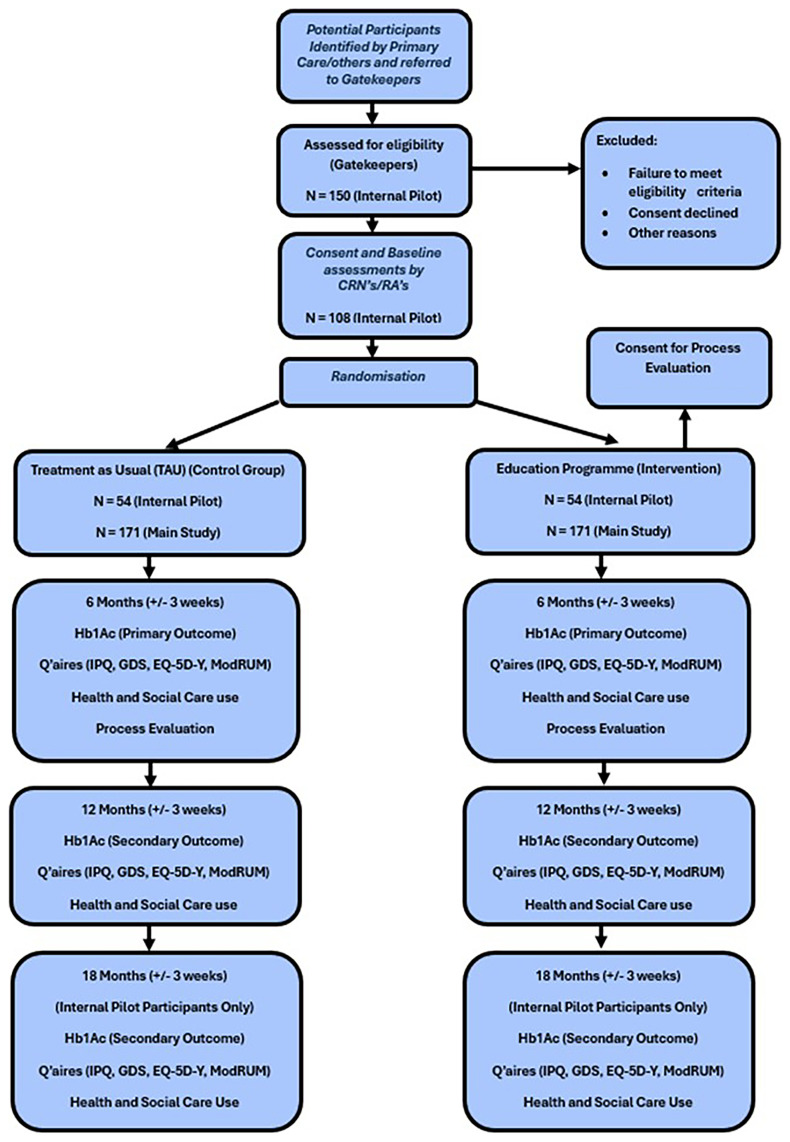
Study Schematic Diagram (Internal Pilot/Main Study).

A range of the secondary outcome measures will be measured to assess group changes and differences over time as specified in Figure 1. These include:

***Body Mass Index (BMI).*** To calculate BMI, height (cm) will be measured to the nearest 0.1cm using Marsden HM-250P Leicester Portable Height Measure. Weight (kg) will be measured to the nearest 0.1kg using Seca 875 Flat Scales.

***Blood sampling.*** Data will be reported on blood lipids (i.e. total cholesterol; low-density lipoprotein (LDL) cholesterol; and high-density lipoprotein (HDL) cholesterol); and estimated Glomerular Filtration Rate (eGFR).

***Blood pressure.*** sSystolic (SBP) and diastolic blood pressure (DBP) will be measured using an OMRON M3 blood pressure monitor. Participants will be asked to rest in a chair for at least 5-minutes before assessment.

***Illness perception measured using the Illness Perception Questionnaire-Revised (IPQ).*** The Illness Perception Questionnaire ([IPQ] ^42^) is a method of assessing cognitive representations of illness. The IPQ is a theoretically derived measure comprising five scales that provides information about the five components that have been found to underlie the cognitive representation of illness. The IPQ will be used to examine a participants’ understanding of diabetes (illness coherence score), perception of the duration of their illness (timeline score) and perception of their ability to affect the course of their diabetes (personal responsibility score).

***Depression measured using the Glasgow Depression Scale.*** The Glasgow Depression Scale for people with a Learning Disability ([GDS-LD] ^43^) is a self-report questionnaire containing 20 items which ask a participant about their experiences in the previous week. Participants must select one from three possible answers: (1) never/no, (2) sometimes and (3) always/a lot, each being scored 0, 1 or 2. Total scores are calculated, ranging from 0 to 40.

***Health related quality of life measured using the EQ-5D-Y.*** This EQ-5D-Y ^44^ is aimed at young people aged 8 years and older and is adapted directly from the EQ-5D-3L with simplified wording making it more appropriate for this population. It is a generic preference-based measure of health, which provides a description of health using five dimensions (mobility, self-care, usual activities, pain/discomfort and anxiety/depression each with 3 levels of severity. Responses can be converted to an overall utility score for the calculation of quality adjusted life years (QALYS).

***Health and social care service use and costs measured using the Modular Resource use Measure (ModRUM).*** ModRUM ^45^ is a validated, concise, generic, measure designed to collect self-report data on the healthcare services people use in UK-based studies. The measure contains a set of core modules that can be expanded to ask participants for additional details by substituting ‘core’ questions for ‘depth’ questions.

### Intervention costs

Intervention costs will be recorded prospectively by the study team.

### Data analysis

The primary analysis will be conducted on all outcome data obtained from all participants as randomised and regardless of protocol adherence, i.e. intention to treat analysis according to their randomisation group. Per protocol analysis will also be carried out: patients who attended all 6 education programme sessions and at least one booster session. A detailed statistical analysis plan (SAP) will be developed. Baseline characteristics and follow-up measurements will be summarized as means and standard deviations, medians and inter-quartile ranges, or numbers and percentages, as appropriate. The difference between randomised groups in change from baseline at 6-months for HbA1c (mmol/mol) levels will be analysed using a t-test. Analysis will use a significance threshold of *p* ≤ 0.05. Secondary analysis for the primary outcome using ANCOVA will adjust for baseline and site. The comparison of other continuous outcomes between the two groups will be investigated using analysis of covariance, adjusting for baseline/other covariates where appropriate or t-test. Dichotomous outcomes will be analysed using chi-square and logistic regression if adjustment for covariates is required. Any adjustment for covariates will be based on clinical input and pre-specified in the SAP. To ensure the quality of the data and trial, we have established a trial steering committee and an independent Data Monitoring Committee which consists of experts external to the trial.

### Health economics evaluation

We will assess the within trial and long-term cost-effectiveness of DESMOND-ID compared with TAU via cost-utility analyses to estimate the cost per QALY. The economic evaluations will be in keeping with the NICE guide to methods of technology appraisal. A National Health Service and personal social services perspective will be adopted and costs and QALYs will be discounted at 3.5% per annum. Further details and full descriptions of analyses will be given in the Health Economics Analysis Plan (HEAP).

### Within trial analysis

Participants’ use of health and social care services will be collected directly from the adults with ID and T2D (with assistance from their carer/partner/advocate) over the study period using diaries and ModRUM questionnaire ^45^. These data collection methods will be piloted before the trial to ensure the language is appropriate for the population and will be completed with carers’ assistance if necessary. Standard unit costs will be used to cost resources. The resource use to deliver DESMOND-ID will be measured prospectively by the study team so the cost per participants can be estimated. Participants’ health-related quality of life will be measured using the EQ-5D-Y over the study period. The UK adult tariff will be used to obtain utilities for the calculation of QALYs ^46^. This approach has been used previously in a NIHR Health Technology Assessment funded study with adults with ID ^32^.

### Long-term economic modelling

We will conduct a model based economic analysis to calculated long-term costs and QALYs of DESMOND-ID versus TAU. To do this we will adapt our existing model of interventions for adults with T2D, the School for Public Health Research Type 2 Diabetes Treatment (SPHR-T2D) model ^47^ so that it is applicable to a population with T2D and mild or moderate ID. The SPHR-T2D treatment model is an individual level microsimulation model, which simulates the life course of people with T2D, modelling trajectories of four metabolic risk factors, namely HbA1c, BMI, SBP, DBP, LDL and HDL. The occurrence of micro vascular (neuropathy, nephropathy, retinopathy), macro vascular (myocardial infarction, stroke, ischaemic heart disease, heart failure), peripheral vascular disease, breast cancer, colorectal cancer, depression and death are estimated in each year for each individual based upon published risk functions, metabolic trajectories (primarily related to the four risk factors), history of prior events, and other characteristics (e.g. diabetes duration). Costs and utility losses are associated with each event included in the model. To adapt the model, firstly, we will conduct literature reviews of existing economic models for people with diabetes or ID, utility parameters and costing studies. Based on these reviews, the data from the trial and discussions with clinical experts in the research team, we will update our model to reflect a population with mild or moderate ID/T2D.

The adapted model will also incorporate data from the trial to estimate the differences in costs and QALYs for people receiving DESMOND-ID versus TAU. We will obtain any treatment related costs from the economic data collected in the trial. We will include any effects of DESMOND-ID compared to TAU on HbA1c, BMI, SBP, DBP, LDL or HDL from the main 12-month trial analysis. How long these effects will be maintained will be informed by the 18-month follow-up for the participants in the internal pilot only (N= 108) and clinical expert opinion. Finally, we will use the baseline data from the trial as far as possible to inform the characteristics of the individuals in the model. Further details and full descriptions of analyses will be given in the HEAP.

### Process evaluation

A process evaluation will be carried out by an independent research team to identify facilitators, barriers and mechanisms involved in the delivery of DESMOND-ID. We will collect process evaluation data using qualitative and quantitative methods. More specifically, we will collect data using interviews with participants in the DESMOND-ID group and educators, and from the trial management logs. We will measure the fidelity of the intervention by observing a sample of the delivered sessions across sites (see Table 2)

**Table 2.** Summary of process evaluation dimensions and data collected.

		Elements measured	Data source
**Implementation constructs evaluated**	**Reach**	The number of intended individuals that participate in the intervention.	Study records
	**Fidelity**	The extent to which the intervention was delivered as planned.	Observations
	**Dose delivered**	The quantity of each intervention component delivered (i.e. educators trained, sessions delivered, participants attended each session).	Study records (attendance lists)
**Implementation constructs evaluated**	**Recruitment**	Success of methods used to approach and recruit participants.	Study Records
	**Exposure**	Proportion of participants in the intervention group who participated in the study.	Study records
	**Context**	Factors external to the intervention, which may influence intervention implementation	Interviews with educators and participants
	**Satisfaction**	Satisfaction of participants with the overall intervention	Interviews with participants

### Observations of DESMOND-ID training and delivery sessions

Intervention fidelity (IF) will explore whether the intervention was delivered as intended. The fidelity of the intervention will be measured through observing a proportion of intervention sessions delivered by educators. We will observe all educators at least once across the different sites and observe a sample of sessions (e.g. carer session at start, weeks 2–7 participant sessions, two booster sessions at 1 and 3 months after the last weekly session). The DESMOND-ID educators will have received training to understand the philosophy of the self-management programme and to learn the content of the sessions (how to deliver in an interactive way), applying facilitation skills based on Motivational Interviewing (techniques including open-ended questions, affirmations, reflections, and summaries). To ensure these skills are practiced consistently across the sites, these will be observed and measured using a bespoke IF checklist and coding manual.

Guided by the recent IF guidance on how to develop IF tools for complex interventions ^48^, the IF tools (checklists and coding manual) will be developed by the Leicester research team, bespoke to the educators’ trained behaviours, key messages of the intervention and duration of the sessions ^49^. Each IF dimension (adherence, delivery and duration) will be measured in the IF checklist as ‘Present’ (the behaviour/message was observed regularly), ‘Absent’ (the behaviour/message was not observed) or ‘Attempted’ (the behaviour/message was observed occasionally). The coding manual will be developed to support the use of the IF checklists, and to ensure that coders measuring fidelity are guided by the same manual. Duration of the group sessions and talk time (the proportion of time the educators and participants talk) will be recorded. To ensure reliability and validity of the IF checklist and coding manual, during the development of the IF tools, inter-rater reliability will be assessed across coders to confirm a satisfactory degree of agreement. A sample of delivered sessions (equivalent of one course) will be observed and coded by two independent coders according to a priori criteria based on the DESMOND-ID training plan and behaviours. In line with this, process variables such as facilitator attendance and duration of session will also be captured. Educators will be given an opportunity at the end of each training session to complete a training evaluation form. Fidelity of training will be assessed quantitatively by calculating the proportion of the presence of pre-specified content (i.e. % planned components as per the training plan).

### Qualitative data collection

Post intervention an evaluation qualitative study (consisting of 1–1 interviews or focus groups) will be conducted at across England, Northern Ireland and Scotland with adults with ID/T2D who participate in the study, and the educators delivering the DESMOND-ID programme. The aim is to explore individual experiences with intervention participants, as well as barriers and facilitators to intervention delivery and participation. The semi-structured interviews will address dimensions of the Normalisation Process Theory ([NPT] ^50^) for the process evaluation, including intervention fidelity, dose delivered, exposure and satisfaction. Educators and participants (from the intervention group) will be invited to participate in this qualitative study at local site level.

### Topic guide

Interviews will be guided by flexible topic guides to cover questions around reasons for taking part, views on intervention, experience in attending and experience in making healthy lifestyle changes. The topic guides will have further input by PPI members with ID, to ensure the questions are appropriate for the target population.

### Participant eligibility and recruitment

Approximately 30–45 adults with ID/T2D across the clinical sites will be recruited for the interviews in the main study. An easy-read invitation letter, an easy-read PIS and reply slip about the interviews will be sent to adults with ID/T2D who participated in the DESMOND-ID education programme, and who agreed to be invited for interview during the consenting process for the main study. They will be asked to return the reply slip by post, or to telephone the research team to express their interest in taking part, or if they would like to ask further information about the qualitative study. The research team will telephone those who are interested in taking part, confirm they are still willing to take part and if they are, they will arrange a date and time for the interview. All educators involved in the delivery of the DESMOND-ID education programme will be invited to take part in a 1:1 or a focus group interview.

### Data collection

To ensure that we purposefully include a wide range of adults with ID/T2D, we will use maximum variation sampling by gender and age. Data collection for this qualitative study will occur shortly after the adults with ID/T2D have attended their second booster session. Each interview is likely to take up to 60 minutes, with regular breaks included if necessary. It will be facilitated by an experienced qualitative researcher and if possible, a co-moderator in each site. We aim to minimise participant burden by taking regular breaks where needed. To aid memory, visuals of the sessions/resources will be shown during the interview to accompany the questions. Interviews with study participants will be conducted in person. All identifiers will be anonymised, and participant confidentiality will be protected. Personal details of participants will be kept confidential and anonymised and will be securely stored in a locked filing cabinet or on secure password protected networks at our research facility adhering to relevant data protection and GDPR policies.

### Data coding and analysis

Transcripts will be imported into qualitative data indexing software NVivo (Lumivero, Version 14) to organise and assist with analysis. Data analysis will begin after the first few interviews to allow time to refine the interview guides accordingly. The data from each participant group will be analysed separately and then compared and reported. Data will be analysed using inductive thematic analysis by the qualitative research team. Interpretation of the findings will be conducted by the qualitative research team.

## Conclusion

DESMOND is a theoretically underpinned intervention using both psychological and educational theories and has been robustly tested and shown to be both clinically and cost-effective for adults with T2D in the general population. Earlier research by Taggart *et al.* ^23^ successfully adapted DESMOND for adults with ID/T2D and demonstrated that this new adapted DESMOND-ID programme can be tested within a definitive trial. This is such a trial and it is the first randomised trial of a DSME programme for adults with ID and T2D, and will be one of the largest trials undertaken in this population.

## Trial status

Protocol version v4.0, 18/01/24. Recruitment started in January 2023 and is projected to complete in September 2025. Data collection commenced in January 2023 and will continue through to November 2026.

## List of abbreviations

**Table:** 

T2D	Type 2 diabetes
ID	Intellectual disability
RCT	Randomised-controlled trial
HbA1c	Glycated haemoglobin
TAU	Treatment as usual
IDF	International Diabetes Federation
WHO	World Health Organisation
NICE	National Institute for Health and Care Excellence
DSME	Diabetes self-management education
DESMOND	Diabetes Education and Self-Management for Ongoing and Newly Diagnosed
NIHR	National Institute for Health and Care Research
PIS	Participant information sheet
PPI	Patient and public involvement
NICTU	Northern Ireland Clinical Trials Unit
BMI	Body Mass Index
LDL	Low-density lipoprotein
HDL	High-density lipoprotein
eGFR	estimated Glomerular Filtration Rate
SBP	Systolic blood pressure
DBP	Diastolic blood pressure
IPQ	Illness Perception Questionnaire
GDS-LD	Glasgow Depression Scale for people with a Learning Disability
QALYS	Quality adjusted life years
ModRUM	Modular Resource use Measure
SAP	Statistical analysis plan
HEAP	Health Economics Analysis Plan
SPHR-T2D	School for Public Health Research Type 2 Diabetes Treatment
IF	Intervention fidelity
NPT	Normalisation Process Theory

## Declarations

### Ethics approval and consent to participate

This study will be conducted in accordance with the guidelines laid down in the Declaration of Helsinki. Ethical approval was received by the Office for Research Ethics Northern Ireland (ORECNI: 22/NI/0156), and research governance was obtained from all participating health boards in Northern Ireland, Scotland and England. Before enrolment, prospective participants will be forwarded adapted user-friendly participant information sheets that have been developed in accordance with our PPI partners, including adults with ID. Research associates and clinical research nurses will be required to check for knowledge and understanding to ensure participants are able to provide informed consent. All participants will be required to sign written informed consent to take part in the study upon enrolment.

KK has acted as a consultant, speaker or received grants for investigator-initiated studies for Abbott, Astra Zeneca, Bayer, Novo Nordisk, Sanofi-Aventis, Servier, Lilly and Merck Sharp & Dohme, Boehringer Ingelheim, Oramed Pharmaceuticals, Pfizer, Roche, Daiichi-Sankyo, Applied Therapeutics, Embecta and Nestle Health Science.

### Consent for publication

Not applicable - no identifying images or other personal or clinical details of participants are presented here or will be presented in reports of the trial results.

## Data Availability

No data are associated with this article. No extended data are associated with this article. Figshare: Spirit Checklist for the My Diabetes & Me: study protocol for a randomised controlled trial to test the clinical and cost-effectiveness of a diabetes self-management education programme for adults with intellectual disabilities ^51^. Available at: https://doi.org/10.6084/m9.figshare.29459048.v1 Data are available under the terms of the Creative Commons Attribution 4.0 International license (CC-BY 4.0).
