# Supplementary material for: My Diabetes & Me: study protocol for a randomised controlled trial to test the clinical and cost-effectiveness of a diabetes self-management education programme for adults with intellectual disabilities
Source: NIHR Open Res. 2025 Oct 6;5:93. [Version 1] doi: 10.3310/nihropenres.13964.1 (PMC12754366; doi:10.3310/nihropenres.13964.1)
Supplement: Peer review history

# Reviewer report: Reviewer response for version 1

**DOI:** 10.3310/nihropenres.15180.r38421

**Published:** 2025-12-30

**Authors:** Tshifhiwa Mukheli

**Affiliations:** 1 Gauteng Department of Health, Johannesburg, South Africa

This protocol plans a two-step trial to test DESMOND-ID, a diabetes education programme for people with intellectual disabilities. It has a clear purpose, strong design, and good involvement from patients. Small changes are needed for recruitment, follow-up, and data plans. Overall, it is well-designed and very important for diabetes care for the vulnerable population 

 1. Is the rationale for, and objectives of, the study clearly described?

**Yes.** The background provides a strong rationale, and objectives are clearly stated and measurable.

 2. Are sufficient details of the methods provided to allow replication by others?

**Yes**. The protocol includes comprehensive methodological details, though SAP and HEAP could include more specifics on missing data handling and subgroup analyses.

 3. Are the datasets clearly presented in a useable and accessible format?

**Not applicable **at this stage, as no datasets are associated with the protocol; however, future data sharing plans should be clarified.

 Overall, the study is well-designed and highly important for improving diabetes care among vulnerable populations.

 Some additional input:

 1. Please define the acronym “DESMOND” fully at the start for clarity.

 2. The sentence *“However, some find this difficult and diabetes self-management education (DSME) programme”* is incomplete and unclear. Consider revising it for clarity and grammatical correctness

Is the study design appropriate for the research question?

Yes

Is the rationale for, and objectives of, the study clearly described?

Yes

Are sufficient details of the methods provided to allow replication by others?

Yes

Are the datasets clearly presented in a useable and accessible format?

Not applicable

Reviewer Expertise:

diabetic foot and its complications, including the knowledge of the healthcare and patients in prevention of diabetic foot ulcers.

I confirm that I have read this submission and believe that I have an appropriate level of expertise to confirm that it is of an acceptable scientific standard.

# Reviewer report: Reviewer response for version 1

**DOI:** 10.3310/nihropenres.15180.r37648

**Published:** 2025-11-10

**Authors:** Betul Hatipoglu

**Affiliations:** 1 University Hospitals Diabetes and Metabolic Care Center, Cleveland, USA; 2 Medicine, Case Western Reserve University School of Medicine (Ringgold ID: 12304), Cleveland, Ohio, USA

The study emphasizes the importance of education in diabetes management. Today, diabetes is worldwide affecting some areas of the world, 20-25 percent of the population, and others, 10 to 12 percent. Nevertheless, it's a significant health crisis, and education is the primary intervention that can really improve the outcomes. Using techniques in adults with learning disabilities becomes even more critical in a disease that relies on self-management. I highly encourage this study to be indexed.

Is the study design appropriate for the research question?

Yes

Is the rationale for, and objectives of, the study clearly described?

Yes

Are sufficient details of the methods provided to allow replication by others?

Yes

Are the datasets clearly presented in a useable and accessible format?

Yes

Reviewer Expertise:

diabetes and population health

I confirm that I have read this submission and believe that I have an appropriate level of expertise to confirm that it is of an acceptable scientific standard.
